# Supplementary material for: Spatial Variation in Stream-Water Bacterial Communities Across Sites Differing in Wild Chinese Giant Salamander Detection Frequency
Source: Biology (Basel). 2026 Sep 18;15(18):1647. doi: 10.3390/biology15181647 (PMC13604818; doi:10.3390/biology15181647)
Supplement: Supplementary file 1 [file biology-15-01647-s001.zip › Data Related to “Spatial Variation in Stream-Water Bacterial Communities across Sites Differing in Wild Chinese Giant Salamander Detection Frequency”/Microbial Data/report/src/hyperlinks/Methods_QIIME1_CH_LRS.pdf]

中文版 Method

仅供客户在文章写作时参考，分析内容和方法请以结题报告

为准， 请客户自行承担文章查重等相关风险。

1 实验流程

1.1 DNA 检测

根据样本类型，进行DNA提取，将提取后的DNA 通过 1%琼脂糖凝胶电泳（120V， 30min）进行质量检测，评估其条带完整性和核酸纯度。将符合要求的 DNA 样本用无菌无核酸酶水稀释至终浓度 1ng/μL，用于后续实验。

1.2 PCR 产物的获取

以稀释后的基因组 DNA 为模板，采用带有特异性条形码（barcode）的引物对目标 16S rRNA 基因区域进行 PCR 扩增。所 有 PCR 混 合 液 加 入 12.5 μL KAPAHiFi HotStart ReadyMix、2.5 μM 引物和10 ng基因组DNA模板， 在95℃下进行3分钟的第一次变性，然后在98℃ (20s)、57℃ (30s) 和 72℃ (75s) 下进行22次循环， 最后在72℃下保持5分钟。扩增产物经 2%琼脂糖凝胶电泳验证后，使用磁珠纯化试剂（如 AMPure XP）去除引物二聚体。

1.3 Kinnex 文库构建

将不同样本的barcode 标记 PCR 产物等摩尔混合，采用 Kinnex 长读长测序技术进行文库构建，具体步骤如下：

末端修复：使用 Kinnex 末端修复酶（20℃，30min）处理 PCR 产物，确保DNA 末端平整化。

接头连接：加入 Kinnex 特异性测序接头（20μM）及 T4 DNA 连接酶（20℃，15min），使 DNA 片段与测序适配体连接。

线性阵列组装：通过 Kinnex 组装酶（37℃，1h）将 16S rRNA 基因片段串联成长片段，形成线性 DNA 阵列。

文库纯化：采用SMRTbell®磁珠进行片段筛选（0.45×和 0.8×磁珠比例），保留 500–2000bp 的目标片段，以提高文库质量。

1.4 文库质量评估与测序

纯化后的文库使用Qubit® 4.0 荧光定量仪测定浓度，并通过 Agilent 2100 检测片段大小分布，确保主峰位于预期范围（1.5–3kb）。符合质量标准的文库在 Revio 测序平台上进行单分子实时（SMRT）测序，以获得高质量的长读长测序数据。。

2 生物信息分析

2.1 数据质量控制

2.1.1 数据拆分

根据 Barcode 序列和 PCR 扩增引物序列从下机数据中拆分出各样本数据。

2.1.2 数据质控

使用 dada2 （R3.5）对拆分获得的 hifireads 经过长度过滤处理得到高质量的数据（Clean Tags）。

2.2 OTU 聚类和物种注释

2.2.1 OTU 聚类

利用Uparse 算法(Uparse v7.0. 1001, http://www.drive5.com/uparse/)(Edgar RC et al., 2013)对所有样本的全部 Effective Tags 进行聚类，默认以 97%的一致性(Identity)将序列聚类成为 OTUs (Operational Taxonomic Units)，同时会选取 OTUs 的代表性序列，依据其算法原则，筛选的是OTUs 中出现频数最高的序列作为 OTUs 的代表序列，用于后续的物种注释。

2.2.2 物种注释

16S: Silva 138.2 数据库（http://www.arb-silva.de/)(Quast C et al.,2012)；

分类注释算法：Mothur。

18S: Silva 138.2 数据库（http://www.arb-silva.de/)(Quast C et al.,2012)；

分类注释算法：RDP。

ITS：Unite v10.0 数据库（https://unite.ut.ee/)(Herr JR et al.,2014)； 分类注释算法：blast

非常规区域：默认用 Micro_NT 数据库（利用NT 库提取古菌、真菌、病毒、细菌整理得到的子库）注释。

备注：

1. 由于 Silva 官网序列文件SILVA_138.2_SSURef_NR99_tax_silva.fasta 仅整理物种名称，缺失物种相关层级信息，同时 Silva 官网提供的层级信息文件不完整，因此需要通过物种名称去 ncbi 获取层级。步骤是先使用 Silva 官网的层级信息补充物种层级，对未定位到物种层级的物种信息则使用 ncbi 官网提供的 dmp 文件进行层级补充。

2. Micro_NT 库注释原理：将序列与 Micro_NT 数据库中的序列进行 blast 比对， 获取该序列与数据库中各序列打分前 20 的结果，按照 bit score 最大值进行筛选，随后使用

LCA 算法来推断该序列所属的最近共同祖先。NT 数据库中大量的未分类信息，导致注释效果大大降低，注释的结果会出现较多的未分类 Unclassfie。为保证数据的精确性，我们在进行最近公共祖先算法获取物种信息时，会忽略 Unclassfied 未分类物种， 降低其对注释造成的影响。

2.2.3 构建系统发育树

使用 MUSCLE (Edgar RC et al.,2004)(Version 3.8.31,http://www.drive5.com/muscle/) 软件进行快速多序列比对，得到所有 OTUs 代表序列的系统发生关系。

2.2.4 数据均一化

最后对各样本的数据进行均一化处理，以样本中数据量最少的为标准进行均一化处理，后续的 Alpha 多样性分析和 Beta 多样性分析都是基于均一化处理后的数据。

2.2.5 物种丰度统计

根据每个样本在不同分类等级（门、纲、目、科、属、种） 的丰度前 10 的物种， 通过SVG 函数绘制Perl 中相对丰度的分布直方图。

2.2.6 热图

利用每个分类级别的的丰度前 35 的物种丰度信息绘制热图，直观地显示了不同的丰度和分类群聚类。这是用 R 的 pheatmap()函数实现的。

2.2.7 三元相图

根据每个分类级别的前 10 个分类群的三元图可以用来显示三个样本之间的丰度差异。它是用 R 的 vcd()函数中计算的。

2.2.8 恩图和花瓣图

Venn 和Flower 图直观地显示了不同样本或组之间的共同和独特信息。Venn 图和Flower 图分别用 VennDiagram() 函数在 R 中生成，用 SVG 函数在 perl 中生成。

2.2.9 系统进化分析

系统发育树，也称为进化树，可以描述不同物种之间的进化关系。选择样本中丰度最高的 100 个属，进行序列比对，用 perl 绘制 SVG 格式的系统发育树。

2.3 样本复杂度分析（**Alpha Diversity**）

2.3.1 Alpha 多样性指数分析

使用 Qiime 软件（Version 1.9.1）计算 Observed-otus, Chao1, Shannon, Simpson, ace, goods- coverage, PD_whole_tree 指数, 使用R 软件 (Version 4.0.3) 绘制稀释曲线。

计算菌群丰度(Community richness) 的指数有：

Chao -the Chao1 estimator (http://www.mothur.org/wiki/Chao)；

ACE the ACE estimator (http://www.mothur.org/wiki/Ace)。

计算菌群多样性 (Community diversity) 的指数有：

Shannon - the Shannon index (http://www.mothur.org/wiki/Shannon)；

Simpson- the Simpson index (http://www.mothur.org/wiki/Simpson)。

计算测序深度的指数有：

Coverage - the Good’s coverage (http://www.mothur.org/wiki/Coverage)。

计算系统发育多样性的指数有：

PD_whole_tree-PD_whole_tree index

(http://scikit-bio.org/docs/latest/generated/skbio.diversity.alpha.faith_pd.html?highlight=pd#skbio.diver sity.alpha.faith_pd)。

2.3.2 物种累积箱型图

为了评估微生物群落的丰富度和样本量。物种积累箱图可以用于可视化，这是用 R 包执行的。

2.3.3 等级梯度曲线

通过观察曲线的宽度和形状来反映样本物种的丰富度和均匀度。这可以使用R 中的 ColorBrewer 软件包绘制。

2.3.4 稀释曲线

测序数量不足可能导致样本信息不足，而过多的测序深度也可能导致不必要的成本增加。因此，确定合适的测序量是至关重要的。绘制稀疏曲线提供了发现序列深度是否足够的可行性的能力。 这是通过使用 plyr 包的 R 来实现的。

2.4 多样本比较分析（Beta Diversity）

2.4.1 Beta 多样性分析

为了评估群落组成的复杂性并比较样本(组)之间的差异，用 Qiime 软件(Version 1.9.1)基于的加权和非加权距离进行β多样性分析。

2.4.2 Beta 多样性热图

β多样性分析用于评估样本在物种复杂性方面的差异。通过 QIIME 软件计算加权 和未加权 unifrac 的β多样性距离。然后绘制一个热图来显示样本之间的 unifrac 距离，这是用Perl 实现的。

2.4.3 UPGMA 聚类分析

基于加权 unifrac 距离矩阵，在UPGMA 上构造了聚类树。这在生态学中被广泛用 于进化分类。UPGMA 图是通过Qiime 中的 UPGMA.tre 函数绘制的。

2.4.4 降维分析

主成分分析（PCA），该分析用于使用带有 R 软件的 ade4 软件包和 ggplot2 软件包（4.0.3 版）来降低原始变量的维数。

主坐标分析（PCoA）用于从复杂和多维数据中获取主坐标并进行可视化。在转换到一组新的正交轴之前，获得了样本之间加权或未加权均匀性的距离矩阵，通过该矩阵，第一主坐标表示最大变化因子， 第二主坐标表示第二大变化因子。PCoA 分析通过 R 软件（ 4.0.3 版） 中 的 ade4 包和 ggplot2 包计算和绘制。

非度量多维度分析（NMDS）来降低数据维度。与 PCoA 类似，NMDS 也使用距离矩阵，但它强调的是数值秩。图上样本点之间的距离只能反映秩信息， 而不能反映数值差异。NMDS 分析是通过带有ade4 软件包和ggplot2 软件包的 R 软件实现的。

2.5 群落差异分析

通过 Anosim、Adonis、MRPP、Simper、T 检验、Metagenomeseq、LEfSe Wilcoxon 秩和检验和Kruskal-Wallis 秩和检验等一系列统计分析，揭示了群落结构的分化。

Anosim、Adonis 和MRPP 分析是分析高维数据组之间差异的非参数检验。这可以分析分组之间的差异是否显著大于组内的差异，这可以确定分组是否有意义。这些可以在 R 软件内使用 vegan 包和 ggplot2 包进行分析和绘制。

Wilcox 秩和检验（Wilcoxon rank-sum test），也叫曼-惠特尼U 检验（Mann–Whitney U test），是两组独立样本非参数检验的一种方法。其原假设为两组独立样本来自的两总体分布无显著差异，通过对两组样本平均秩的研究来实现判断两总体的分布是否存在差异，该分析可以对两组样本的物种进行显著性差异分析，并对P 值可以进行多种方法的校正。

Kruskal-Wallis H test，简称克氏秩和检验，它是一种将两组独立样本的 Wilcox 秩和检验推广到多组（大于等于 3）独立样本非参数检验的方法。该分析可以对多组样本的物种进行显著性差异分析。

Simper 可以揭示每个物种对群体之间分化的贡献。选出了前 10 个物种，并将其显示在图表上。这在R 中使用Vegan 软件包和 ggplot2 软件包进行。

Metagenomeseq 可以展示群体之间表现出显著差异的物种。在 R 中使用

MetagenomeSeq 包进行。

LEfSe 被广泛用于发现生物标志物，它可以揭示宏基因组特征。这是 lefse 的单独软件进行计算和绘制的。

2.6 功能预测

PICRUSt（V1.1.4）主要用于预测基于标记基因的宏基因组功能。PICRUSt2（V2.3.0）是 PICRUSt 的改进版本。

Tax4Fun（V0.3.1）是一个 R 软件，广泛用于肠道和土壤样本。总的来说，与 PICRUSt 相比，它可以提供更准确的结果，尤其是对于土壤样本。

BugBase 工具，可以发现显微组织的表型。它可以根据七种表型对微生物群落进行分类：

革兰氏阳性、革兰氏阴性、生物膜形成、致病性、含移动元素、氧气利用（包

括好氧、厌氧和可培养厌氧）和氧化应激耐受性。

在处理真菌样本时，可以使用FunGuild 工具，对主要的营养类型进行分类，研究具体的真菌功能分类。

FAPROTAX 是主要阐明生物化学过程和元素时发挥重要作用的软件。

2.7 关联分析

2.7.1 网络图

为了探索物种之间的共生关系，揭示环境因素对群落结构的影响，绘制了二维和三维网络图进行可视化。

2.7.2 环境因子分析

可以使用 spearman 相关性检，CCA/RDA 和 dbRDA 等进一步分析来反映环境因素与物种丰度之间的相关性。所有这些图表和分析都是在 R 中完成的。

2.8 群落构建机制

iCAMP、βNTI 和 NST 是微生物生态学中用于分析群落构建机制（即驱动微生物群落组成变化的生态过程）的常用指标，它们在解析群落多样性、环境筛选、随机性与确定性过程的作用等方面各有侧重。

3 参考文献

Bokulich NA, Subramanian S, Faith JJ, et al. Quality-filtering vastly improves diversity estimates from Illumina amplicon sequencing.Nature Methods. 2012;10(1):57-59. doi:10.1038/nmeth.2276.

Edgar RC, Haas BJ, Clemente JC, Quince C, Knight R. UCHIME improves sensitivity and speed of chimera detection. Bioinformatics. 2011;27(16):2194- 2200. doi:10.1093/bioinformatics/btr381.

Edgar RC. MUSCLE: multiple sequence alignment with high accuracy and high throughput.Nucleic Acids Research. 2004;32(5):1792- 1797. doi:10. 1093/nar/gkh340.

Edgar RC. UPARSE: highly accurate OTU sequences from microbial amplicon reads.Nature Methods. 2013;10(10):996-998. doi:10. 1038/nmeth.2604.

Herr JR, Qbik M, Hibbett DS. Towards the unification of sequence-based classification and sequence-based identification of host-associated microorganisms.New Phytologist. 2014;205(1):27- 31. doi:10.1111/nph.13180.

Magoc T, Salzberg SL. FLASH: fast length adjustment of short reads to improve genome assemblies.Bioinformatics. 2011;27(21):2957-2963. doi:10. 1093/bioinformatics/btr507.

Quast C, Pruesse E, Yilmaz P, et al. The SILVA ribosomal RNA gene database project: improved data processing and web-based tools.Nucleic Acids Research. 2012;41(D1):D590- D596. doi:10.1093/nar/gks1219.
